# Plethora of Resistance Genes in Carbapenem-Resistant Gram-Negative Bacteria in Greece: No End to a Continuous Genetic Evolution

**DOI:** 10.3390/microorganisms10010159

**Published:** 2022-01-13

**Authors:** Katerina Tsilipounidaki, Zoi Athanasakopoulou, Elke Müller, Sindy Burgold-Voigt, Zoi Florou, Sascha D. Braun, Stefan Monecke, Nikolaos K. Gatselis, Kalliopi Zachou, Aggelos Stefos, Ilias Tsagalas, Marina Sofia, Vassiliki Spyrou, Charalambos Billinis, George N. Dalekos, Ralf Ehricht, Efthymia Petinaki

**Affiliations:** 1 Faculty of Medicine, University of Thessaly, 41500 Larissa, Greece; tsilipou@uth.gr (K.T.); zflorou@uth.gr (Z.F.); ngatsel@uth.gr (N.K.G.); zachouk@uth.gr (K.Z.); evanstef1@uth.gr (A.S.); tsagalas@outlook.com (I.T.); dalekos@uth.gr (G.N.D.); 2 Faculty of Veterinary Science, University of Thessaly, 43100 Karditsa, Greece; zathanas@uth.gr (Z.A.); msofia@uth.gr (M.S.); billinis@uth.gr (C.B.); 3 Leibniz Institute of Photonic Technology (IPHT), 07745 Jena, Germany; elke.mueller@leibniz-ipht.de (E.M.); sindy.burgold-voigt@leibniz-ipht.de (S.B.-V.); sascha.braun@leibniz-ipht.de (S.D.B.); stefan.monecke@leibniz-ipht.de (S.M.); Ralf.Ehricht@leibniz-ipht.de (R.E.); 4 InfectoGnostics Research Campus, 07743 Jena, Germany; 5 Institut fuer Medizinische Mikrobiologie und Hygiene, Universitätsklinikum Dresden, 01307 Dresden, Germany; 6 Faculty of Animal Science, University of Thessaly, 41110 Larissa, Greece; vasilikispyrou@uth.gr; 7 Faculty of Public and One Health, University of Thessaly, 43100 Karditsa, Greece; 8 Institute of Physical Chemistry, Friedrich Schiller University Jena, 07737 Jena, Germany

**Keywords:** carbapenem resistance, antimicrobial resistance genes, *Klebsiella pneumoniae*, *Acinetobacter baumannii*, *Pseudomonas aeruginosa*, Greece

## Abstract

Carbapenem-resistant Gram-negative bacteria are a public health threat that requires urgent action. The fact that these pathogens commonly also harbor resistance mechanisms for several other antimicrobial classes further reduces patient treatment options. The present study aimed to provide information regarding the multidrug resistance genetic background of carbapenem-resistant Gram-negative bacteria in Central Greece. Strains from a tertiary care hospital, collected during routine practice, were characterized using a DNA microarray-based assay. Various different resistance determinants for carbapenems, other beta-lactams, aminoglycosides, quinolones, trimethoprim, sulfonamides and macrolides were detected among isolates of the same sequence type. Eighteen different multidrug resistance genomic profiles were identified among the twenty-four *K. pneumoniae* ST258, seven different profiles among the eight *K. pneumoniae* ST11, four profiles among the six *A. baumannii* ST409 and two among the three *K. oxytoca*. This report describes the multidrug resistance genomic background of carbapenem-resistant Gram-negative bacteria from a tertiary care hospital in Central Greece, providing evidence of their continuous genetic evolution.

## 1. Introduction

The dissemination of carbapenem-resistant (CR) Gram-negative bacteria, including *Klebsiella pneumoniae*, *Acinetobacter baumannii* and *Pseudomonas aeruginosa*, has dramatically increased over the last years [1]. Infections caused by these microorganisms are linked with prolonged time of hospitalization leading to increased healthcare costs as well as with elevated mortality rates [2]. Detailed knowledge of the characteristics of these pathogens is essential for the development of novel antibiotics and potential new therapeutic targets [3].

Two main resistance mechanisms against carbapenems in enterobacteria are known: ampC overexpression accompanied by a porin loss [4,5] and transmissible genes encoding carbapenemases [6]. The corresponding genes and alleles are usually located on plasmids as well as other mobile genetic elements (MGEs) [7]. Plasmids with carbapenemase genes often additionally harbor toxin–antitoxin systems which prevent plasmid loss even in the absence of selective pressure caused by antibiotics [8]. Furthermore, the capacity of these bacteria to survive in the nosocomial environment helps them to acquire genetic elements from other bacteria, which include novel antibiotic-resistance determinants or pathogenicity genes [9].

Recent reports showed an increasing prevalence of CR Gram-negative bacteria and their rapid worldwide spread. The four most prevalent carbapenemase genes are *bla*_KPC_, *bla*_NDM_, *bla*_OXA-48_ and *bla*_VIM_ [6]. Infections caused by CR Gram-negative bacteria are usually difficult to treat [10]. Treatment options are limited since carbapenemase genes are often co-localized on mobile genetic elements together with additional resistance genes conferring resistance to aminoglycosides and/or fluoroquinolones. Therefore, only a few antibiotics remain effective, such as colistin, fosfomycin and tigecycline, as well as, in some cases, the monobactam aztreonam, which is not hydrolyzed by metallo-beta-lactamases (e.g., VIM and NDM) [11].

As early as 2009 the US Centers for Disease Control and Prevention (CDC) recommended an active screening as a prerequisite for specific quarantine arrangements that might help to prevent the dissemination of carbapenem-resistant pathogens [12,13]. Several other governmental institutions and agencies such as the World Health Organization (WHO), the European Centre for Disease Prevention and Control (ECDC) and the US Agency for Healthcare Research and Quality (AHRQ) also shared this view [14,15,16,17].

In Greece, the rate of CR Gram-negative bacteria is among the highest worldwide [18,19,20,21]. Given that the detection of different resistance genes and MGEs is costly and time-consuming, no data from our country are available regarding the characterization of the whole genetic background of these pathogens. The purpose of the present study was the detection of a plethora of resistance genes in a representative collection of CR Gram-negative bacteria, using the microarray-based CarbDetect AS-2 Kit (Abbott, Jena, Germany).

## 2. Materials and Methods

### 2.1. Selection of the CR Gram-Negative Isolates

The study was conducted in the University Hospital of Larissa (UHL), a tertiary care 600-bed hospital in the Thessaly region (Central Greece) which serves a population of approximately 1,000,000 inhabitants. Based on the UHL surveillance protocol, all CR bacteria are routinely tested for carbapenemase-encoding genes, are subjected to multi-locus sequence typing (MLST) and are stored at −80° for epidemiological purposes. Identification and susceptibility testing of all CR strains are performed using the automated system BD Phoenix™ M50. The detection of carbapenemase-encoding genes (*bla*_KPC_, *bla*_NDM_, *bla*_VIM_, *bla*_OXA-like_) and MLST typing are performed as previously described [22].

A total of 44 CR Gram-negative isolates (6 *Acinetobacter baumannii*, 3 *Pseudomonas aeruginosa* and 35 *Klebsiella* spp.) were selected from the collection of routine isolates as described above. The inclusion of the bacteria into the study was based on the type of carbapenemase they produced, their sequence type, and their antibiotic susceptibility profiles, so as to include as many different profiles for each sequence type as possible. All strains were isolated from clinical samples between January 2019 and April 2020.

### 2.2. Molecular Characterization

A molecular characterization of the selected strains was performed using the CarbDetect AS-2 Kit (Abbott, Jena, Germany), according to the manufacturer′s instructions, as previously described [23]. The kit detects a total of 134 genes including 111 genes and alleles associated with resistance to carbapenems, cephalosporins, aminoglycosides, fluoroquinolones, trimethoprim, sulfonamides and macrolides, as well as 10 genes encoding multidrug efflux pumps and toxin–antitoxin systems (Table 1). The Result Collector 2.0 (Abbott, Jena, Germany) was used to automatically summarize the results obtained from the microarray analysis.

## 3. Results

The group of 44 carbapenem-resistant strains that were selected for analysis consisted of 32 *K. pneumoniae*, six *A. baumannii*, three *Klebsiella oxytoca* and three *P. aeruginosa*.

Thirty-three of the selected isolates harbored one carbapenemase gene and eleven isolates harbored two. Among *K. pneumoniae* strains *bla*_KPC_ was the most commonly identified carbapenemase gene, found in 24 out of the 32 isolates. *bla*_NDM_ was detected in eight isolates, while *bla*_VIM_ was only detected in five and in all cases co-existed with *bla*_KPC_. All *A. baumannii* strains harbored a *bla*_OXA-23_-like gene, whereas all the *K. oxytoca* and all the *P. aeruginosa* harbored *bla*_VIM_. Variant *bla*_VIM-2_ was specifically identified in a single *P. aeruginosa* isolate.

Genes responsible for ESBL and broad-spectrum beta-lactamases’ production were detected in 40 out of the 44 carbapenem-resistant strains. The gene *bla*_SHV_ was identified in 28 *K. pneumoniae* isolates and in two *K. oxytoca*, *bla*_CTX-M-1/15_ in 21 *K. pneumoniae*, *bla*_TEM_ in 13 *K. pneumoniae* and in four *A. baumannii*, *bla*_VEB_ in four *K. pneumoniae*, *bla*_OXA-1_ in 16 *K. pneumoniae* and in two *P. aeruginosa*, *bla*_OXA-9_ in two *K. pneumoniae* and *bla*_OXA-6_ in one *K. pneumoniae*. AmpC genes were detected in four isolates; two *K. pneumoniae* harbored *bla*_ACT_ and two *K. oxytoca* harbored *bla*_MOX-CMY9_.

Aminoglycoside resistance genes were present in 41 out of the 44 carbapenem-resistant strains. Among *K. pneumoniae*, the combination of genes *aac(3′)-Ia*, *aac(6′)-Ib*, *aadA1*, and *aphA* was detected in six isolates, the combination *aac(6′)-Ib*, *strA*, and *strB* in four, the *aac(6′)-Ib*, *aadA1*, and *aadA2* in three, the *aac(6′)-Ib*, *aadA2*, and *aphA* in three, the *aac(6′)-Ib* and *aadA2* in three, the *aadA1*, *aadB*, *ant2*, *aphA*, *strA*, and *strB* in two, the *aadA1*, *aadB*, *ant2*, *rmtB*, *strA*, and *strB* in two, the *aac(6′)-Ib*, *aadA2*, *aphA*, *strA*, and *strB* in one and the *aadA1*, *aphA*, *strA*, and *strB* in one. Four *K. pneumoniae* only possessed *aac(6′)-Ib* and one only *aphA*. Additionally, five out of the six *A. baumannii* isolates harbored aminoglycoside resistance genes. Four co-harbored the *aphA*, *armA*, *strA*, and *strB* and one the *aac(3′)-Ia*, *aadA1*, *armA*, *strA*, and *strB*. Regarding the three *K. oxytoca*, the *aac(6′)-Ib*, *aac(6′)-IIc*, *aadA2*, *aphA*, *strA*, and *strB* genes were detected in two strains and the *aac(6′)-Ib*, *aac(6′)-IIc*, *aphA*, and *strB* genes in one isolate. Concerning the *P. aeruginosa* isolates, one harbored the combination *aac(6′)-Ib*, *aadA1*, *strA*, and *strB*, while one only harbored the *aac(6′)-Ib* and one the *aac(6′)-Iic*. Overall, *aac(6′)-Ib* was the most common gene, found in 29 out of the 44 CR strains.

Plasmid-mediated quinolone resistance (PMQR) genes were identified in seven strains. In particular, gene *qnrS* was detected in four *K. pneumoniae* and in the three *K. oxytoca*.

Genes associated with trimethoprim resistance were detected in 23 *K. pneumoniae* and in the three *K. oxytoca*. Fifteen *K. pneumoniae* harbored *dfrA14*, 10 *dfrA12* and four *dfrA1*. *DfrA14* and *dfrA12* co-existed in six isolates. All the *K. oxytoca* harbored *dfrA19*. Regarding sulfonamide resistance genes, these were detected in 27 *K. pneumoniae*, two *A. baumannii*, the three *K. oxytoca* and in two *P. aeruginosa*. *Sul1* was identified in 16 *K. pneumoniae*, one *A. baumannii*, the three *K. oxytoca* and in two *P. aeruginosa*. *Sul2* was detected in 21 *K. pneumoniae*, one *A. baumannii* and two *K. oxytoca*, while *sul3* was present in three *K. pneumoniae*.

Macrolide resistance genes were identified in 16 strains. Ten *K. pneumoniae* harbored *mph* alone (*n* = 3) or in combination with *mrx* (*n* = 7). Additionally, all six *A. baumannii* isolates harbored *mph*.

Genes associated with MGEs were detected in a total of 36 out of the 44 carbapenem-resistant isolates. *intl1* was detected in 29 *K. pneumoniae*, one *A. baumannii*, the three *K. oxytoca* and the three *P. aeruginosa*. Twenty of the *intl1* positive *K. pneumoniae* additionally harbored *tnpISEcp1*.

Finally, the *oqxA* and *oqxB* genes, encoding *oqxAB* efflux pump, were present in 26 *K. pneumoniae*, while the *splA* and *splT* genes, encoding the *SplTA* toxin–antitoxin system, were present in all the six *A. baumannii* isolates.

Overall, 18 distinct genomic profiles were identified among the 24 *K. pneumoniae* ST258, seven distinct profiles among the eight *K. pneumoniae* ST11, four profiles among the six *A. baumannii* ST409 and two among the three untyped *K. oxytoca*.

**Figure 1:**
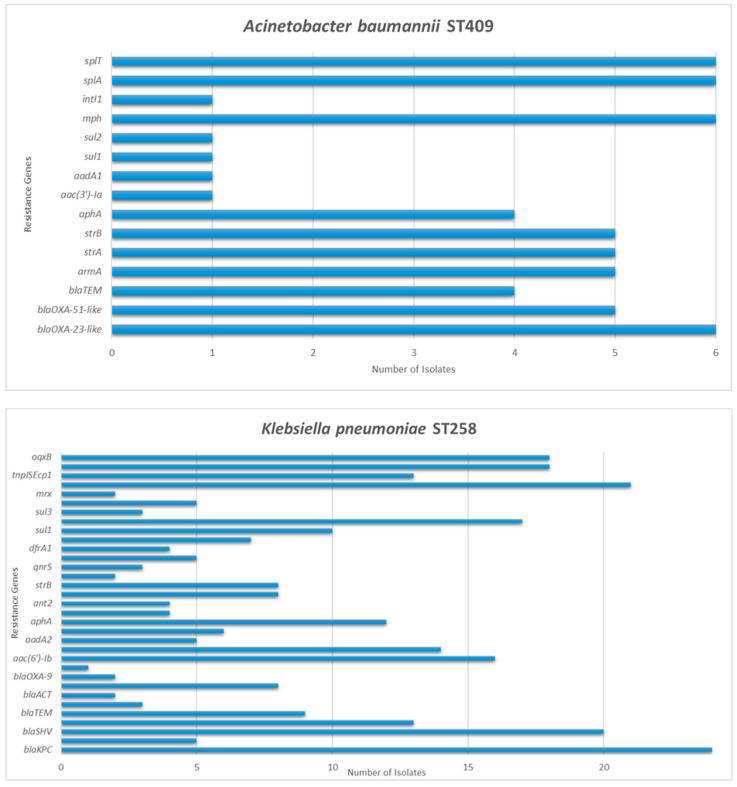

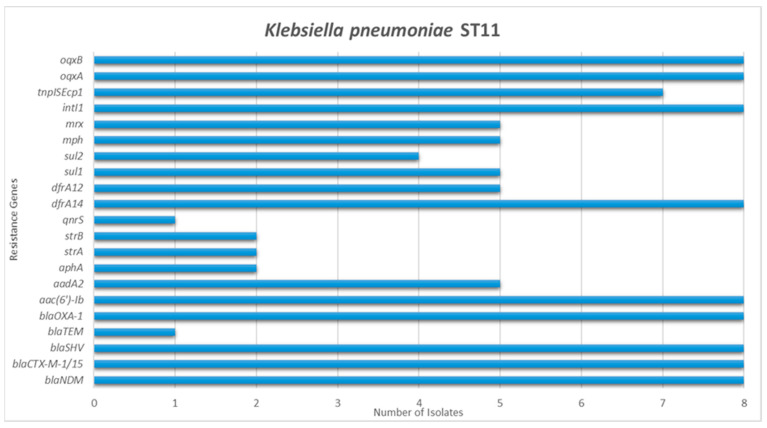
Detection frequency of each resistance gene among *Acinetobacter baumannii* and *Klebsiella pneumoniae* isolates.

The genomic characteristics of the carbapenem-resistant isolates are presented in Table 2 and in Figure 1. The antibiotic susceptibility profiles of the isolates were in concordance with the genotypes.

## 4. Discussion

In recent years, multidrug resistance has evolved to one of the greatest challenges in the health sector, affecting not only hospital settings but also the community, animals and the environment [24,25]. Carbapenem-resistant pathogens represent a threat highly potent to cause outbreaks, while it is anticipated that new unique β-lactamases with unusual properties will be identified in the near future given the widespread presence of β-lactamases genes and the unceasing pressure from the use of β-lactam antibiotics [26,27,28]. The present study aimed to unveil the molecular multidrug resistance determinants of CR Gram-negative bacteria isolated from the University Hospital of Larissa, a hospital that serves the population of Central Greece. A microarray-based assay was selected as the typing tool, as an alternative to whole genome sequencing, since it is a technique suitable for screening research, excellent in specificity and sensitivity [29].

The majority of *K. pneumoniae* strains in our study expressed carbapenem resistance due to carriage of *bla*_KPC_. Carbapenemases of the KPC family have the most extensive global distribution of all carbapenemases that are associated with Enterobacteriaceae and are highly prevalent in Mediterranean countries, especially Italy and Greece [30]. Despite the fact that Greece used to be the epicenter of VIM-producing Enterobacteriaceae [31], these did not predominate, underlining the fast evolution in the molecular epidemiology of carbapenemases, as has previously been illustrated by Galani et al. [32]. Coexistence of OXA-23-like and TEM was the primary resistance profile in the *A. baumannii* isolates, as has previously been described in China [33]. The oxacillinase *bla*_OXA-23_-like is also amongst the most dominant resistance genes that have been reported in *A. baumannii* from Germany [34]. All the *P. aeruginosa* harbored *bla*_VIM_, which was expected considering the pre-existing data from the region [20].

Genes associated with aminoglycoside resistance were detected in 41 strains. Aminoglycosides are usually part of the empirical treatment of serious nosocomial infections in most Greek tertiary hospitals and constitute one of the few remaining options in the battle against CR pathogens. That could explain and drive the wide dissemination of the respective resistance genes. The *aac(6′)-Ib* was the most common gene detected in this study. Former studies have also stated its frequent co-occurrence with carbapenemases genes in Switzerland [35], Spain [36], and India [37], as well as Greece [38].

Trimethoprim/sulfamethoxazole resistance genes *sul* and *dfrA* were detected in 24 strains. *DfrA14* was the most common trimethoprim resistance gene, which is in agreement with a recent study from South Africa [39]. Concerning sulfonamide resistance genes, *sul2* predominated, which is in contrast with former findings from Brazil [40]. *Sul2* variant has, however, also been detected in high rates among carbapenemase-producing *K. pneumoniae* strains isolated from intensive care unit patients in Turkey [41].

Concerning quinolone resistance genes, the plasmid-encoded gene *qnrS* was detected in seven strains; six harbored *qnrS* and possessed *bla*_VIM_ alone (*n* = 3) or in combination with *bla*_KPC_ (*n* = 3), while the remaining one possessed *bla*_NDM_. The presence of genes *oqxA* and *oqxB* might also have contributed to the fluoroquinolone resistance profile of 26 *K. pneumoniae*. The plasmidic efflux pump OqxAB confers resistance to multiple agents, including fluoroquinolones as well as biocides, and has been shown to play a role in the selection of fluoroquinolone resistance in different *K. pneumoniae* clones [42,43].

One of the main drivers for the recorded rapid dispersion of multidrug resistance is the presence of MGEs [44]. In our study, *intl1* was the only integrase gene detected among the CR strains, while the *intl2* and *intl3* genes were not present in any isolate. These findings are in concordance with earlier reports about KPC-2 positive *K. pneumoniae* from a pediatric hospital in China [45]. In Southern Brazil, though, class 2 integrons were more frequently detected than class 1 among OXA-23 *A. baumannii* [46]. Class I integrons are known to harbor various antimicrobial resistance gene cassettes encoding β-lactamases, *dfr* and *sul* variants, qacEΔ1 (quaternary ammonium compound disinfectant), as well as aminoglycoside-modifying enzymes [47]. This probably explains the genotypic profile of the *intl1* positive strains that we examined, which presented different combinations of resistance determinants for at least three classes of antimicrobials. Furthermore, we detected the *ISEcp1* element, known to be implicated in the mobilization of AMR genes such as *bla*_CTX-M_ and *bla*_KPC_ [48,49]. The resistance determinants identified in isolates that were tested positive for *tnpISEcp1* are subsequently considered more likely to be disseminated horizontally via *ISEcp1*-mediated transposition among the same or different bacterial species.

Finally, genes *splA* and *splT*, encoding the plasmid borne *SplTA* toxin–antitoxin system, were identified in all the CR *A. baumannii* isolates of our study. The *SplTA* is widely spread in the *A. baumannii* plasmidome, including carbapenem-resistant clinical isolates, and can act as a plasmid stabilization and maintenance mechanism even in the absence of antimicrobial selective pressure. It is also involved in the successful transmission of plasmids carrying carbapenemase genes, favoring even further their dissemination [50].

In conclusion, according to our findings, strains that belonged to the same MLST clone had different molecular resistance patterns, indicating a potential continuous genetic evolution of antimicrobial resistance. The ability of bacteria to evolve their AMR characteristics might continue to undermine health care, economic development, and life expectancy if infection control measures are not implemented.

## Figures and Tables

**Table 1:** Genes and alleles detected by the CarbDetect AS-2 Kit, per category of genes.

Category of Genes	Genes and Alleles
Carbapenemases	*bla*_BIC_, *bla*_DIM_, *bla*_GES_, *bla*_GIM_, *bla*_GOB_, *bla*_IMI-3_ (*nmcA*), *bla*_IMI-R_, *bla*_IMP_, *bla*_IMP-25_ (*bla*_SIM-1_), *bla*_IMP-35_, *bla*_IND_, *bla*_KHM_, *bla*_KPC_, *bla*_NDM_, *bla*_PAM-1_, *bla*_SFH-1_, *bla*_SMB-1_, *bla*_SME_, *bla*_SPM-1_, *bla*_TMB-1_, *bla*_VIM_, *bla*_VIM-2_, *bla*_VIM-7_, *bla*_OXA-23_-like, *bla*_OXA-40_-like, *bla*_OXA-48_-like, *bla*_OXA-51_-like, ISA*ba1* to *bla*_OXA-51_, no ISA*ba1* to *bla*_OXA-51_, *bla*_OXA-54_, *bla*_OXA-55_, *bla*_OXA-58_, *bla*_OXA-134/235/284_, *bla*_OXA-143/182/253/255_, *bla*_OXA-181/232_, *bla*_OXA-214_, *bla*_OXA-279_, *bla*_OXA-292_
ESBL	*bla*_CME_, *bla*_CTX-M-1/15_, *bla*_CTX-M-2_, *bla*_CTX-M-8_, *bla*_CTX-M-9_, *bla*_PER-1_, *bla*_PER-2_, *bla*_SHV_, *bla*_TEM_, *bla*_VEB_, *bla*_OXA-18_, *bla*_OXA-45_
AmpC	*bla*_MIR_, *bla*_ACC_, *bla*_ACT_, *bla*_CMY_, *bla*_DHA_, *bla*_FOX_, *bla*_MOX_, *bla*_MOX-CMY9_
Other Beta-lactamases	*bla*_OXA-1_, *bla*_OXA-2_, *bla*_OXA-9_, *bla*_OXA-10_, *bla*_OXA-60_
Aminoglycoside Resistance	*aac(3′)*, *aac(3′)-Ia*, *aac(3′)-Ib*, *aac(3′)-Ic*, *aac(3′)-Ie*, *aac(3′)-Iva*, *aac(6′)*, *aac(6′)-31*, *aac(6′)-Ib*, *aac(6′)-II*, *aac(6′)-Iia*, *aac(6′)-Iic*, *aac*-*aph*, *aadA1*, *aadA2*, *aadA4*, *aadB*, *ant2*, *aphA*, *armA*, *grm*, *npmA*, *rmtA*, *rmtB*, *rmtC*, *rmtD*, *strA*, *strB*
Quinolone Resistance	*qepA*, *qnrA1*, *qnrB*, *qnrC*, *qnrD*, *qnrS*
Trimethoprim Resistance	*dfrA1*, *dfrA12*, *dfrA13*, *dfrA14*, *dfrA15*, *dfrA17*, *dfrA19*, *dfrA5*, *dfrA7*
Sulfonamide Resistance	*sul1*, *sul2*, *sul3*
Macrolide Resistance	*mdh*, *mrx*
Markers for Mobile Genetic Elements	*intI1*, *intI2*, *intI3*, *tnpISEcp1*
Multidrug Efflux Pumps	*oqxA*, *oqxB*
Toxin–Antitoxin Systems	*higA*, *higB*, *splA*, *splT*

**Table 2:** Genomic characterization of the carbapenem-resistant isolates.

Strain	Species	MLST Typing	Carbapenemase Genes	ESBL Genes	AmpC Genes	Other Beta-Lactamase Genes	Genes Associated with Aminoglycoside Resistance	Genes Associated with Quinolone Resistance	Genes Associated with Trimethoprim Resistance	Genes Associated with Sulfonamide Resistance	Genes Associated with Macrolide Resistance	Genes Associated with Mobile Genetic Elements	Genes Associated with a Multidrug Efflux Pump	Genes Encoding a Toxin–Antitoxin System
A114-1	*A. baumannii*	ST409	*bla*_OXA-23_-like, *bla*_OXA-51_-like	-	-	-	*aac(3′)-Ia*, *aadA1*, *armA*, *strA*, *strB*	-	-	*sul1*	*mph*	*intI1*	*-*	*splA*, *splT*
A90-2	*A. baumannii*	ST409	*bla*_OXA-23_-like, *bla*_OXA-51_-like	*bla*_TEM_	-	-	*aphA*, *armA*, *strA*, *strB*	-	-	-	*mph*	-	-	*splA*, *splT*
A261-2	*A. baumannii*	ST409	*bla*_OXA-23_-like, *bla*_OXA-51_-like	*bla*_TEM_	-	-	*aphA*, *armA*, *strA*, *strB*	-	-	-	*mph*	-	-	*splA*, *splT*
A262-2	*A. baumannii*	ST409	*bla*_OXA-23_-like, *bla*_OXA-51_-like	*bla*_TEM_	-	-	*aphA*, *armA*, *strA*, *strB*	-	-	-	*mph*	-	-	*splA*, *splT*
A265	*A. baumannii*	ST409	*bla*_OXA-23_-like, *bla*_OXA-51_-like	*bla*_TEM_	-	-	*aphA*, *armA*, *strA*, *strB*	-	-	*sul2*	*mph*	-	-	*splA*, *splT*
A268	*A. baumannii*	ST409	*bla*_OXA-23_-like	-	-	-	*-*	-	-	-	*mph*	-	-	*splA*, *splT*
A1793	*K. oxytoca*	-	*bla*_VIM_	-	-	-	*aac(6′)-Ib*, *aac(6′)-IIc*, *aphA*, *strB*	*qnrS*	*dfrA19*	*sul1*	-	*intI1*	-	-
A1829	*K. oxytoca*	-	*bla*_VIM_	*bla*_SHV_	*bla*_MOX-CMY-9_	-	*aac(6′)-Ib*, *aac(6′)-IIc*, *aadA2*, *aphA*, *strA*, *strB*	*qnrS*	*dfrA19*	*sul1*, *sul2*	-	*intI1*	-	-
A1846	*K. oxytoca*	-	*bla*_VIM_	*bla*_SHV_	*bla*_MOX-CMY-9_	-	*aac(6′)-Ib*, *aac(6′)-IIc*, *aadA2*, *aphA*, *strA*, *strB*	*qnrS*	*dfrA19*	*sul1*, *sul2*	-	*intI1*	-	-
A1795	*K. pneumoniae*	ST258	*bla*_KPC_	*bla*_TEM_	-	-	*aac(6′)-Ib*, *aadA1*, *aadA2*	-	*dfrA12*	*sul2*, *sul3*	-	*intI1*	-	-
A1821	*K. pneumoniae*	ST258	*bla*_KPC_	*bla*_CTX-M-1/15_	-	-	*aac(3′)-Ia*, *aac(6′)*, *aac(6′)-Ib*, *aadA1*, *aphA*	-	-	*sul1*, *sul2*	-	*intI1*, *tnpISEcp1*	-	-
A1869	*K. pneumoniae*	ST258	*bla*_KPC_	*bla*_CTX-M-1/15_, *bla*_SHV_	-	-	*aac(3′)-Ia*, *aac(6′)*, *aac(6′)-Ib*, *aadA1*, *aphA*	-	-	*sul1*, *sul2*	-	*intI1*, *tnpISEcp1*	-	-
A1833	*K. pneumoniae*	ST258	*bla*_KPC_, *bla*_VIM_	*bla*_SHV_, *bla*_TEM_, *bla*_VEB_	-	*bla*_OXA-1_	*aadA1*, *aadB*, *ant2*, *aphA*, *strA*, *strB*	*qnrS*	*dfrA1*	*sul1*, *sul2*	*mph*	*intI1*	-	-
A1839	*K. pneumoniae*	ST258	*bla*_KPC_	*bla*_CTX-M-1/15_, *bla*_SHV_, *bla*_TEM_	-	-	*aac(6′)-Ib*, *strA*, *strB*	-	*dfrA14*	*sul2*	-	*intI1*, *tnpISEcp1*	*oqxA*, *oqxB*	-
A1841	*K. pneumoniae*	ST258	*bla*_KPC_	*bla*_CTX-M-1/15_, *bla*_SHV_	-	-	*aac(3′)-Ia*, *aac(6′)-Ib*, *aadA1*, *aphA*	-	-	*sul1*, *sul2*	-	*intI1*, *tnpISEcp1*	-	-
A1845	*K. pneumoniae*	ST258	*bla*_KPC_, *bla*_VIM_	*bla*_SHV_	-	-	*aadA1*, *aphA*, *strA*, *strB*	*qnrS*	*dfrA1*	*sul1*, *sul2*	*mph*	*intI1*	*oqxA*, *oqxB*	-
A1847	*K. pneumoniae*	ST258	*bla*_KPC_, *bla*_VIM_	*bla*_CTX-M-1/15_	-	*bla*_OXA-1_	*aac(6′)-Ib*	-	*dfrA14*	-	-	*intI1*, *tnpISEcp1*	*oqxA*, *oqxB*	-
A1850	*K. pneumoniae*	ST258	*bla*_KPC_, *bla*_VIM_	*bla*_CTX-M-1/15_, *bla*_SHV_	-	*bla*_OXA-1_	*aac(6′)-Ib*	-	*dfrA14*	-	-	*intI1*, *tnpISEcp1*	*oqxA*, *oqxB*	-
A1875	*K. pneumoniae*	ST258	*bla*_KPC_, *bla*_VIM_	*bla*_SHV_, *bla*_TEM_, *bla*_VEB_	-	*bla*_OXA-1_	*aadA1*, *aadB*, *ant2*, *aphA*, *strA*, *strB*	*qnrS*	*dfrA1*	*sul1*, *sul2*	*mph*	*intI1*	-	-
A1881	*K. pneumoniae*	ST258	*bla*_KPC_	*bla*_CTX-M-1/15_	-	-	*aphA*	-	*dfrA1*	*sul1*	-	*intI1*, *tnpISEcp1*	*oqxA*, *oqxB*	-
A1871	*K. pneumoniae*	ST258	*bla*_KPC_	*bla*_SHV_	-	*bla*_OXA-6_	*aac(6′)-Ib*	-	-	-	-	-	*oqxA*, *oqxB*	-
A10-1	*K. pneumoniae*	ST258	*bla*_KPC_	*bla*_CTX-M-1/15_, *bla*_SHV_, *bla*_TEM_	-	*bla*_OXA-1_	*aac(6′)-Ib*, *strA*, *strB*	-	*dfrA14*	*sul2*	-	*intI1*, *tnpISEcp1*	*oqxA*, *oqxB*	-
A41-1	*K. pneumoniae*	ST258	*bla*_KPC_	*bla*_SHV_, *bla*_TEM_, *bla*_VEB_	*bla*_ACT_	*bla*_OXA-1_	*aadA1*, *aadB*, *ant2*, *rmtB*, *strA*, *strB*	-	*dfrA14*	*sul2*	-	*intI1*	*oqxA*, *oqxB*	-
A50-1	*K. pneumoniae*	ST258	*bla*_KPC_	*bla*_SHV_	-	-	*-*	-	-	-	-	-	*oqxA*, *oqxB*	-
A99-1	*K. pneumoniae*	ST258	*bla*_KPC_	*bla*_SHV_	-	-	*-*	-	-	-	-	-	*oqxA*, *oqxB*	-
A55-1	*K. pneumoniae*	ST258	*bla*_KPC_	*bla*_CTX-M-1/15_, *bla*_SHV_, *bla*_TEM_	-	*bla*_OXA-1_, *bla*_OXA-9_	*aac(6′)-Ib*, *aadA2*, *aphA*, *strA*, *strB*	-	*dfrA12*, *dfrA14*	*sul2*	*mph*, *mrx*	*intI1*, *tnpISEcp1*	*oqxA*, *oqxB*	-
A56-1	*K. pneumoniae*	ST258	*bla*_KPC_	*bla*_SHV_, *bla*_TEM_	-	*bla*_OXA-9_	*aac(6′)-Ib*, *aadA2*, *aphA*	-	*dfrA12*	*sul1*	*mph*, *mrx*	*intI1*	*oqxA*, *oqxB*	-
A72-1	*K. pneumoniae*	ST258	*bla*_KPC_	*bla*_SHV_, *bla*_TEM_	-	-	*aac(6′)-Ib*, *aadA1*, *aadA2*	-	*dfrA12*	*sul2*, *sul3*	-	*intI1*	*oqxA*, *oqxB*	-
A90-1	*K. pneumoniae*	ST258	*bla*_KPC_	*bla*_SHV_, *bla*_TEM_	-	-	*aac(6′)-Ib*, *aadA1*, *aadA2*	-	*dfrA12*	*sul2*, *sul3*	-	*intI1*	*oqxA*, *oqxB*	-
A91-1	*K. pneumoniae*	ST258	*bla*_KPC_	*bla*_CTX-M-1/15_, *bla*_SHV_	-	-	*aac(3′)-Ia*, *aac(6′)-Ib*, *aadA1*, *aphA*	-	-	*sul1*, *sul2*	-	*intI1*, *tnpISEcp1*	*oqxA*, *oqxB*	-
A105-1	*K. pneumoniae*	ST258	*bla*_KPC_	*bla*_CTX-M-1/15_, *bla*_SHV_	-	-	*aac(3′)-Ia*, *aac(6′)-Ib*, *aadA1*, *aphA*	-	-	*sul1*, *sul2*	-	*intI1*, *tnpISEcp1*	*oqxA*, *oqxB*	-
A126-1	*K. pneumoniae*	ST258	*bla*_KPC_	*bla*_CTX-M-1/15_, *bla*_SHV_	-	-	*aac(3′)-Ia*, *aac(6′)-Ib*, *aadA1*, *aphA*	-	-	*sul1*, *sul2*	-	*intI1*, *tnpISEcp1*	*oqxA*, *oqxB*	-
A264	*K. pneumoniae*	ST258	*bla*_KPC_	*bla*_CTX-M-1/15_, *bla*_SHV_	*bla*_ACT_	*bla*_OXA-1_	*aadA1*, *aadB*, *ant2*, *rmtB*, *strA*, *strB*	-	*dfrA14*	*sul2*	-	*intI1*, *tnpISEcp1*	*oqxA*, *oqxB*	-
A24-1	*K. pneumoniae*	ST11	*bla*_NDM_	*bla*_CTX-M-1/15_, *bla*_SHV_	-	*bla*_OXA-1_	*aac(6′)-Ib*	*qnrS*	*dfrA14*	*sul2*	-	*intI1*, *tnpISEcp1*	*oqxA*, *oqxB*	-
A97-1	*K. pneumoniae*	ST11	*bla*_NDM_	*bla*_CTX-M-1/15_, *bla*_SHV_	-	*bla*_OXA-1_	*aac(6′)-Ib*, *aadA2*, *aphA*	-	*dfrA12*, *dfrA14*	*sul1*, *sul2*	*mph*, *mrx*	*intI1*, *tnpISEcp1*	*oqxA*, *oqxB*	-
A100-1	*K. pneumoniae*	ST11	*bla*_NDM_	*bla*_CTX-M-1/15_, *bla*_SHV_, *bla*_TEM_	-	*bla*_OXA-1_	*aac(6′)-Ib*, *strA*, *strB*	-	*dfrA14*	*sul2*	-	*intI1*	*oqxA*, *oqxB*	-
A102-1	*K. pneumoniae*	ST11	*bla*_NDM_	*bla*_CTX-M-1/15_, *bla*_SHV_	-	*bla*_OXA-1_	*aac(6′)-Ib*, *aadA2*, *aphA*	-	*dfrA12*, *dfrA14*	*sul1*	*mph*, *mrx*	*intI1*, *tnpISEcp1*	*oqxA*, *oqxB*	-
A198	*K. pneumoniae*	ST11	*bla*_NDM_	*bla*_CTX-M-1/15_, *bla*_SHV_	-	*bla*_OXA-1_	*aac(6′)-Ib*, *strA*, *strB*	-	*dfrA14*	*sul2*	-	*intI1*, *tnpISEcp1*	*oqxA*, *oqxB*	-
A261-1	*K. pneumoniae*	ST11	*bla*_NDM_	*bla*_CTX-M-1/15_, *bla*_SHV_	-	*bla*_OXA-1_	*aac(6′)-Ib*, *aadA2*	-	*dfrA12*, *dfrA14*	*sul1*	*mph*, *mrx*	*intI1*, *tnpISEcp1*	*oqxA*, *oqxB*	-
A261-3	*K. pneumoniae*	ST11	*bla*_NDM_	*bla*_CTX-M-1/15_, *bla*_SHV_	-	*bla*_OXA-1_	*aac(6′)-Ib*, *aadA2*	-	*dfrA12*, *dfrA14*	*sul1*	*mph*, *mrx*	*intI1*, *tnpISEcp1*	*oqxA*, *oqxB*	-
A262-1	*K. pneumoniae*	ST11	*bla*_NDM_	*bla*_CTX-M-1/15_, *bla*_SHV_	-	*bla*_OXA-1_	*aac(6′)-Ib*, *aadA2*	-	*dfrA12*, *dfrA14*	*sul1*	*mph*, *mrx*	*intI1*, *tnpISEcp1*	*oqxA*, *oqxB*	-
A84-1	*P. aeruginosa*	ST235	*bla*_VIM-2_	-	-	*bla*_OXA-1_	*aac(6′)-Ib*, *aadA1*, *strA*, *strB*	-	-	*sul1*	-	*intI1*	-	-
A29-1	*P. aeruginosa*	ST111	*bla*_VIM_	-	-	*bla*_OXA-1_	*aac(6′)-Ib*	-	-	*sul1*	-	*intI1*	-	-
A102-2	*P. aeruginosa*	ST111	*bla*_VIM_	-	-	-	*aac(6′)-Ilc*	-	-	-	-	*intI1*	-	-

## Data Availability

All data generated during this study are presented within the manuscript.
